# Adherence to Exercise and Functional Rehabilitation Programs in Patients with Cardiovascular Diseases: Barriers and Strategies

**DOI:** 10.3390/jcdd13010008

**Published:** 2025-12-22

**Authors:** Gianluca Pagnoni, Aurora Vicenzi, Susan Darroudi, Arianna Maini, Francesco Sbarra, Francesco Marangi, Marco Loffi, Milena Nasi, Marcello Pinti, Valentina Selleri, Alessio Baccarani, Gianluca Carnevale, Carlo Mario Lombardi, Daniela Aschieri, Anna Vittoria Mattioli, Francesco Fedele, Francesca Coppi

**Affiliations:** 1 Cardiology Unit of Emergency Department, Guglielmo da Saliceto Hospital, 29121 Piacenza, Italy; 2 National Institute for Cardiovascular Research (INRC), Via Irnerio 48, 40126 Bologna, Italy; 3 Department of Medical and Surgical Sciences for Children and Adults, University of Modena and Reggio Emilia, Via del Pozzo 71, 41124 Modena, Italy; 4 Cardiology Division, Department of Biomedical Metabolic and Neural Sciences, University of Modena and Reggio Emilia, Via del Pozzo 71, 41124 Modena, Italy; 5 Division of Cardiology, Azienda Socio-Sanitaria Territoriale di Cremona, 26100 Cremona, Italy; 6 Department of Surgery, Medicine, Dentistry and Morphological Sciences, University of Modena and Reggio Emilia, 41125 Modena, Italy; 7 Department of Life Sciences, University of Modena and Reggio Emilia, Via G. Campi 287, 41125 Modena, Italy; 8 Division of Plastic and Reconstructive Surgery, Department of Medical and Surgical Sciences, Modena Policlinico Hospital, University of Modena and Reggio Emilia, 41124 Modena, Italy; 9 Division of Cardiology, Cremona Hospital, 26100 Cremona, Italy; 10 Cardiology, ASST Ospedali Civili di Brescia, Department of Medical and Surgical Specialties, Radiological Sciences, and Public Health, University of Brescia, 25123 Brescia, Italy; 11 Department of Quality of Life, Alma Mater Studiorum—University of Bologna, 40126 Bologna, Italy; 12 Sapienza University of Rome, 00185 Rome, Italy

**Keywords:** cardiac rehabilitation, exercise adherence, gender disparities, cardiovascular disease, rehabilitation barriers, intervention strategies

## Abstract

Adherence to exercise-based cardiac rehabilitation (CR) is essential for preventing and managing cardiovascular disease (CVD). Participation in CR reduces all-cause mortality by 27% and cardiac deaths by 31% and lowers rehospitalization rates while also improving functional capacity and quality of life. However, many patients do not start, complete, or maintain CR, resulting in reduced functional abilities, a higher risk of recurring events, and poorer long-term outcomes. This narrative review summarizes patterns of adherence to exercise and CR in CVD, with a specific focus on sex- and gender-related differences in referral, participation, and completion. We synthesize evidence on biological, psychological, and social barriers that limit engagement and describe emerging strategies, such as technology-enabled and home-based programs, multidisciplinary care, and family-centered models, to enhance adherence. Finally, we propose a practical, gender-aware framework for CR design and delivery that can be adjusted and evaluated across diverse healthcare settings to guide clinical practice and future research.

## 1. Define Adherence and Its Importance in CVD Patients

In academic literature, adherence in cardiovascular rehabilitation (CR) is typically defined as participating in at least 80% of the recommended training sessions, a standard often used to evaluate outcomes [1]. The 80% threshold is a widely adopted benchmark for deeming exercise interventions adherent across various studies. Adherence can also be described as the alignment between a patient’s actions and the agreed-upon exercise regimen, which can be influenced by psychological and sociocultural factors. It includes not only frequency but also adherence to prescribed intensity and the overarching goals of the CR program [1].

Adhering to exercise and CR regimens is essential for improving patient outcomes and supporting ongoing, active lifestyles after cardiac events. Understanding the various factors that influence engagement, such as personalized treatment plans and strong psychological support, is critical to maximizing the effectiveness of CR programs [1]. Unfortunately, evidence shows that adherence to CR programs is often limited, with up to half of patients dropping out within the first few months. Major factors for non-adherence include age, the presence of additional health conditions, socioeconomic challenges, and limited awareness of the benefits of CR [2,3].

Engaging in CR has been linked to a 27% decrease in all-cause mortality and a 31% reduction in cardiac mortality, as well as a reduced risk of rehospitalization [3,4,5], improved quality of life [5], and enhanced cardiac function, while regular exercise boosts functional capacity, mood, and overall well-being [6]. Sustained engagement with therapeutic programs is essential for the success of secondary prevention in CVD. Adhering to exercise-based rehabilitation offers key benefits, including reducing mortality and morbidity and lowering the risk of recurrent cardiovascular events. Addressing participation barriers through comprehensive, personalized care can increase patient engagement and ultimately improve health outcomes. Continuous support throughout the CR journey is vital to maintain adherence and ultimately improve the quality of life and prognosis for these individuals [6].

Furthermore, recent data in patients with systemic sclerosis and pulmonary hypertension show that the TAPSE/sPAP ratio, an echocardiographic, noninvasive index of right ventricle-pulmonary artery coupling calculated as TAPSE (a measure of right ventricular systolic function) divided by sPAP (an estimate of pulmonary arterial systolic pressure/afterload), is a strong independent predictor of cardiovascular events and mortality, underlining how cardiopulmonary functional markers are associated with adverse outcomes and reinforcing the need for highly adherent CR programs [7].

## 2. Prevalence of Adherence to Exercise/Rehabilitation Programs According to Sex in CVD Patients

Men generally exhibit higher adherence to CR [8] programs than women. A meta-analysis reported that CR adherence rates range from approximately 36.7% to 84.6%, with an average of around 66.5%. After a myocardial infarction, women’s participation rates are approximately 36% lower than men’s, and women demonstrate significantly lower completion rates in CR programs [9].

Women comprise a minority of CR participants, about 27.3% in meta-analytic analyses. Female patients show lower referral, participation, and completion rates relative to their male counterparts. Data specific to regions further highlights differences [10]. For example, an 18-year study from Iran reported men making up 73.69% of CR participants [11]. In Germany, adherence to follow-up initiatives after phase III CR averaged 54%, with gender gaps (55% in men vs. 50% in women) [12]. On the other hand, a study from Portugal showed higher engagement, with patients completing an average of 14 CR sessions, which is equivalent to 92% of the planned sessions [9].

Lower adherence among women is associated with worse health outcomes, including a higher risk of recurrent cardiovascular events compared to men. This is influenced by psychosocial factors such as caregiving duties, mental health concerns (depression, anxiety), broader societal norms, educational disparities, and limited access to services. Underrepresentation of women in research also plays a role. The Yentl syndrome describes a pattern where women receive equitable care only when their symptoms resemble those traditionally associated with men [12]. This highlights the importance of targeted interventions to improve adherence to CR among women [12].

The studies showed that women experienced 31% of acute coronary syndrome events, but they only participated in 17.8% of the CR program [13]. This indicates lower referral rates, participation, and completion compared to males [14]. In stroke rehabilitation, completion rates were nearly equal between sexes (women 74.5% vs. men 75.4%; *p* = 0.7) and there was no significant difference in attendance to pre-scheduled sessions (*p* = 0.6). The only notable sex difference occurred in patients younger than 41 years, with 59% of women completing vs. 85% of men (*p* = 0.02) [15].

CR programs are home-based or community-based. Home-based CR shows promising adherence, with a 6-week program achieving an 81.3% completion rate (13 of 16 participants) and an 83.1% adherence to the exercise protocol [16]. Network meta-analyses suggest home-based CR, especially when combined with mobile health interventions, achieves the highest adherence (83.8%). In a phase III community-based CR program, attendance decreased significantly from 73.38% ± 18.09%at three months to 68.14% ± 17.15% at the six-month interval (*p* < 0.001). Among participants who completed 12 months, attendance further declined to 66.8% ± 18.34% at the one-year follow-up [17].

Several studies showed that at one year, adherence varied across behaviors: 98% maintained acceptable adherence to the Mediterranean diet, 83% showed good adherence to physical exercise, and 79% of smokers achieved smoking cessation. On average, 68% of patients achieved good adherence across all three lifestyle components [18].

The largest dropout occurs between program eligibility and initiation. Approximately 36% of eligible patients never engage with CR, and another 26% who do engage fail to initiate. Once the program starts, completion rates are relatively strong at 78.5% [19]. Several factors are associated with higher dropout, including Eastern European nationality, a sedentary lifestyle, and participation in home-based programs (which correlated with lower adherence) [18]. Younger age and being employed in a “blue-collar” occupation showed a tendency toward poorer adherence, though these associations were not statistically significant [18].

## 3. Objectives and Methodology

This narrative review aimed to describe patterns of adherence to exercise-based cardiac rehabilitation (CR) in patients with CVD, examine sex and gender-related barriers and facilitators and summarize strategies to improve adherence. We searched major databases (PubMed/MEDLINE, Embase, Web of Science, Scopus, CINAHL, Cochrane Library) from 2010 to 2025 using terms related to CR, exercise adherence and sex/gender differences.

## 4. Biological and Physiological Factors

### 4.1. Hormonal Influences and Physiological Differences

Sex differences and hormonal influences play a role in the complex patterns of adherence to CR programs [20]. These patterns are influenced by biological factors such as sex hormones, psychological variations in motivation and barriers, and social factors like gender roles and expectations. Understanding these various influences is crucial for developing more inclusive and effective CR programs.

A systematic review of 88 studies examining physiological responses to CR found that men and women respond similarly to most physiological variables, but men show greater benefits in maximal oxygen consumption, functional capacity, six-minute walk distance, and grip strength [20]. On average, women have a lower maximal oxygen uptake (VO2 max) compared to men, partly due to smaller heart size and lower hemoglobin levels that affect oxygen transport [21]. However, women may benefit as much as men, if not more, in terms of mortality reduction [20]. Analyses of CR patients revealed significant sex differences in training responses. Women had lower baseline aerobic capacity across all diagnoses and showed less improvement in directly measured peak oxygen uptake (13% vs. 17% improvement) [22]. Recognizing sex differences in CR responses should inform program design to optimize adherence and outcomes for both sexes. Women-focused CR programs may address specific barriers and preferences, although current evidence shows mixed results compared with traditional mixed-sex programs [23].

One of the other physiological differences between men and women is muscle mass, bone density, and aerobic capacity [24,25,26]. Sex-based differences in body composition influence the results of calorie restriction. Men typically have greater muscle mass and strength than women, largely due to hormonal factors such as testosterone [27]. These differences may contribute to better performance in strength-related tasks and could be a key driver of why men often show larger gains in exercise capacity [22]. Evidence suggests that women can achieve similar relative improvements in strength as men when engaged in strength training, but they generally experience less muscle hypertrophy [28]. However, both genders can experience similar relative improvements in VO2 max through aerobic training [27]. These differences in aerobic capacity suggest that CR programs should be customized for each gender to enhance effectiveness and safety.

Estrogen has multiple protective effects on skeletal muscle through three main pathways: (1) Acting as an antioxidant to limit oxidative damage. (2) Serving as a membrane stabilizer by intercalating into membrane phospholipids. (3) Binding to estrogen receptors to regulate downstream genes and molecular targets. In addition, estrogen stimulates muscle repair and regenerative processes, including the activation and proliferation of satellite cells [20,24,27,28]. Women face a higher risk of osteoporosis, especially after menopause, due to reduced estrogen levels that negatively impact bone health [29]. This underscores the importance of including weight-bearing exercises in women’s rehabilitation programs to maintain bone density. Postmenopausal women may experience exercise-related muscle damage. Clinicians should monitor symptoms, consider gradual progression, incorporate resistance training, and implement recovery strategies to reduce risks [20,27].

### 4.2. Sociodemographic Factors and Family Support

Sociodemographic factors have a significant impact on patients’ adherence to CR programs. Married individuals demonstrate a statistically higher completion rate for phase 2 rehabilitation (*p* = 0.031). Patients with a high school education or less are more likely to adhere to phase 2 sessions (*p* = 0.014) and are more inclined to participate in CR centers. Retired or unemployed patients are more likely to complete phase 2 rehabilitation compared to those who are employed (*p* = 0.06). These findings highlight the importance of considering sociodemographic characteristics when creating strategies to improve engagement and outcomes in CR [30]. The role of social and family support in adherence to exercise and CR programs for patients with CVD has gained prominence in recent studies. While this support often promotes healthier lifestyles and better health outcomes, findings in the available literature show both positive and, in one sample, negative associations. These divergent results likely reflect differences in measurement methods, cultural contexts and potential confounders. A mixed-methods behavioral model demonstrated that perceived social support directly improves exercise adherence, with this effect further augmented through higher exercise self-efficacy and reduced fear of exercise [31].

Gender-related factors play a role in healthcare interactions and treatment adherence. Higher education and clear communication between physicians and patients are linked to better adherence, potentially influenced by sociocultural contexts [12]. Moreover, Social support patterns are different between genders. Women often face challenges balancing caregiving responsibilities that can impact their participation in CR programs [32]. Despite lower participation rates, women may still experience equal or greater mortality benefits from CR [21], although they typically achieve lower metabolic equivalent (MET) scores compared to men after completing rehabilitation [22].

**Figure 1:**
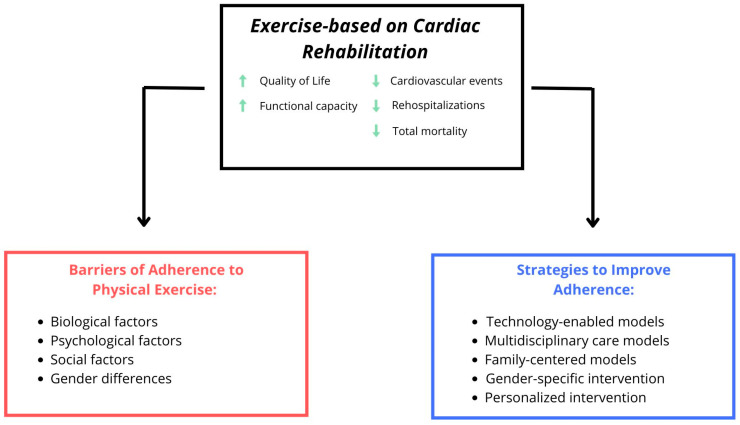
Exercise-based cardiac rehabilitation improves quality of life and prognosis. Adherence is hindered by biological, psychological, social and gender-related barriers, and can be enhanced by technology-enabled, multidisciplinary, family-centered, gender-sensitive and personalized care models.

**Figure 2:**
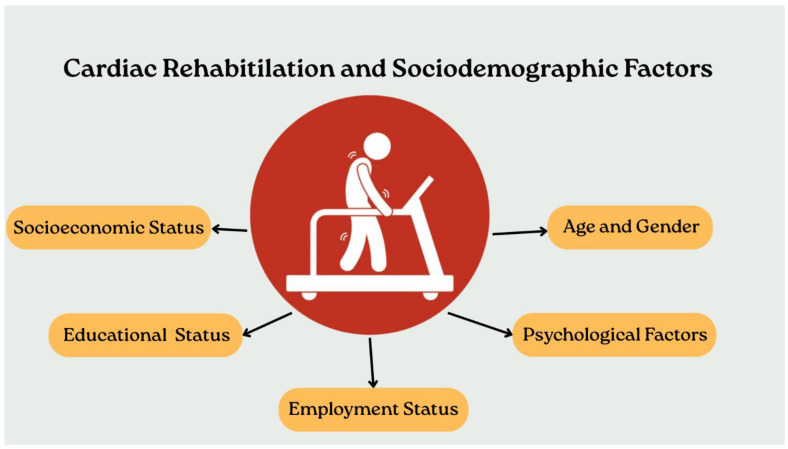
Graphical representation of the main sociodemographic factors that can influence access, adherence, and outcomes of cardiac rehabilitation: socioeconomic status, educational level, employment status, age and gender, and psychological factors.

The long-term benefits of CR, such as reduced mortality and lower recurrence of cardiovascular events, tend to be more favorable for men. Women often experience less positive outcomes following events like acute coronary syndrome, with higher mortality rates reported despite lower event rates before treatment [8]. Across multiple studies, clear positive associations have been found between levels of social support and adherence to self-care behaviors among people with CVD. Social support can be categorized into emotional, instrumental and informational types, all of which significantly influence patients’ motivation to engage in health-promoting behaviors, including exercise and CR [31]. Emotional support, such as encouragement and expressions of care, appears to be particularly impactful in reducing feelings of isolation and depression that often accompany CVD [33]. Figure 1 shows the important factors in cardiac rehabilitation. Key sociodemographic determinants associated with CR participation and adherence are summarized in Figure 2.

Research emphasizes the significant role that family plays in CVD rehabilitation. Families can act as a valuable source of support and encouragement, but they can also contribute to emotional stress. Positive family relationships, characterized by encouragement and active participation in health-related activities, have been shown to improve adherence to CR programs, while negative dynamics can impede recovery [33]. Women often face unique obstacles when it comes to participating in CR, highlighting the potential benefits of having a family-focused support system in place. Participation rates in traditional CR programs are typically lower for women compared to men, underscoring the importance of creating programs that cater to their specific needs and incorporate family support [29]. Family members’ understanding and actively encouraging healthy behaviors can reshape patients’ motivation over time. Shared family experiences in physical activity often correlate with better adherence to CR after a myocardial infarction (MI) [29].

Interviews with patients and their families indicate that adjusting to a new post-MI life frequently requires family backing, which can be crucial in shaping patients’ commitment to physical activity [29]. Families that recognize the importance of exercise provide instrumental support, reinforcing patients’ confidence and willingness to follow exercise regimens [6].

## 5. Barriers to Adherence and Non-Adherence as a Major Challenge

Cardiovascular rehabilitation [34] programs are essential for the secondary prevention and management of CVD. However, non-adherence to these programs, particularly to exercise-based components, undermines their effectiveness [34,35]. Many people discontinue activity early, and gender differences in participation rates persist. Non-adherence emerges as a central component of this challenge, amplifying negative clinical consequences such as mortality, morbidity, hospital exacerbations, and a deterioration in quality of life. Addressing the combined biological, psychological and social influences is crucial to improve CR outcomes for both sexes [14,36,37].

### 5.1. Age-Related Barriers to Exercise Adherence in CVD Patients

Older adults constitute a large percentage of CVD patients and have specific challenges in staying physically active routinely. It has also been demonstrated that an increase in age has a significant negative effect on adherence to exercise habits and is influenced by a number of physiological, psychological, and social factors. These factors should be closely considered for effective intervention programs to facilitate physical activity in elderly people with CVD [38].

### 5.2. Physical Limitations and Comorbidities

Typical physical constraints in older adults with CVD have historically rendered systematic exercise programs less feasible for older adults with CVD. In older patients [39]. A recent study published in 2023 highlights that 68% of CVD patients aged 65 and older identified physical discomfort or comorbidities as primary barriers to exercise [40]. Reduced Muscle Strength and Joint Pain are directly linked with aging and notably affect older adults’ functional capabilities, leading to difficulties in starting or maintaining an exercise program [41]. Moreover, comorbidities like arthritis, diabetes and chronic obstructive pulmonary disease (COPD) also often occur more frequently as people age. This increasing prevalence makes it more challenging to stick to exercise schedules. With a decrease in cardiorespiratory fitness, the risks associated with exercise participation can seem daunting to older adults. This often leads to reluctance in participating in physical activity. Issues such as anxiety and depression can exacerbate physical limitations and further discourage engagement in physical activities, highlighting the need for integrated approaches that address both physical and mental health.

### 5.3. Fear of Injury or Exacerbation of Symptoms

A significant barrier is the fear of injury and the lack of knowledge regarding its benefits of CR and its processes. Many patients are not adequately informed about their condition and do not understand how participating in CR exercises can improve their cardiovascular health and overall well-being [29]. They fear injury or worsening of CVD symptoms, such as chest pain or shortness of breath. This phenomenon, often referred to as kinesiophobia, an excessive fear of movement, leads many older adults to avoid exercise altogether, creating a vicious cycle that hinders physical health management. A recent meta-analysis of 15 studies on patients with heart disease found that kinesiophobia is highly prevalent (approximately 61% of patients) and is strongly associated with anxiety, lower exercise self-efficacy, and lower education and income levels.

### 5.4. Gender Differences in Exercise Adherence Among CVD Patients

Gender plays a significant role in shaping attitudes, behaviors, and barriers related to exercise adherence in CVD patients. Men and women often encounter distinct challenges and motivators, which should be considered when designing interventions.

### 5.5. Perceived Barriers and Motivations

Barriers and motivators are believed to be greater among women with CVD than men and are often perceived as reflecting caregiving duties, insufficient time, less self-efficacy, or other potential pitfalls. A 2020 meta-analysis comparing women and men with CVD showed that 65% of the factors associated with non-adherence in women were family-related compared to 35% in men. Men, however, are more inclined to list work-related stress and lack of interest as barriers. This gender disparity in adherence reflects the complex interplay of societal roles and expectations placed on women, and underscores the importance of addressing gender-based factors in CVD treatment strategies [42].

### 5.6. Psychological Factors

Depression and anxiety seem more common in these women with CVD, and they are negatively associated with exercise adherence, with more females reported to have depression than males. According to a study, women with CVD and comorbid depression were 40% less likely to participate in cardiac rehabilitation when the study participants were compared to men [8]. Conversely, men are more prone to exercise-associated anxiety as it concerns performance issues.

### 5.7. Cultural and Societal Expectations

Traditional gender-based norms of the social sphere play a big role and greatly impact exercise practices. In several places, women are more concerned with child care versus self-care in comparison to a healthy individual, and physical activity participation decreases. On the other hand, when men are under societal pressure from working out harder too, burnout or injury is bound to be a downside as well. Cultural norms, especially about gender, are important for influencing exercise habits. Traditional gender roles in many societies, for example, suggest that women are expected to keep themselves modest and attend to the duties of a family member, often deterring them from engaging in physical activities deemed unfeminine. In the same vein, having the expectation that men should lead competitive and vigorous sports can result in unhealthy exercise behaviors, as men may shy away from moderate or pleasurable activity [43].

Women have unique barriers to physical activity compared with men, research indicates. Segar’s study shows women report more barriers to exercise for themselves, as they believe they have less control over whether or not they engage in physical activity because of social standards, such as childcare and housework [44]. A cultural stereotype may paint some women as inactive or athletic, and as a result, women may experience less physical activity. Women remain at the lower end of the physical activity pyramid compared to men, everywhere around the world, where global statistics indicate that an estimated 33.8% of women fall below the recommended level of activity, and 28.7% of men [45]. This is frequently explained and described not only from biological causes but by systemic socio-cultural and structural ones.

Men being subjected to society’s norms around high-intensity activity are also at risk for unhealthy active behaviors, including more injuries or burnout. Men may also put themselves under pressure to meet the masculine value of toughness and strength, leading them to overexert themselves in physicality [46].

A concise overview of sex-related patterns in referral, perceived barriers, psychosocial factors and outcomes in exercise-based cardiac rehabilitation is summarized in Table 1.

### 5.8. Cost and Insurance Issues

Financial constraints are common, especially for low- and fixed-income patients who may be hesitant to pay out-of-pocket expenses for CR sessions that are not fully covered by insurance. The burden of co-pays and deductibles makes CR less accessible for many individuals, leading to reduced participation rates. Additionally, costs associated with transportation, equipment, or even the CR sessions themselves can discourage participation. Some evidence suggests that patients with Medicaid insurance have a particularly low CR participation rate [47].

### 5.9. Support Systems

Family and social support have been identified as both facilitators and barriers. Supportive family members can boost adherence by encouraging participation, while a lack of support or negative comments can hinder patient motivation and confidence. Peer support within CR programs is helpful, but not all patients have access to a network that promotes their participation [48].

Qualitative studies and surveys, consistently highlight caregiving and family responsibilities as significant obstacles to exercise and CR participation particularly for women. International research consistently demonstrates that family obligations decrease attendance, enrollment, and interfere with the time and energy required for recommended exercise [49].

In settings where women are expected to prioritize household roles and family harmony, illness can threaten their identity, leading women to deprioritize self-care activities such as exercise [48]. Caregiving creates role conflict that undermines exercise and participation in CR [49]. Among women enrolled in CR, family responsibilities were explicitly identified as one of the top barriers to session adherence, alongside distance and transportation, in a large multi-country assessment [50]. Mixed-methods data indicate that women in caregiving roles are statistically less likely to participate in physical activity programs, with family responsibilities reported by a large proportion of participants [51]. In some cultural groups (e.g., South Asian Punjabis), women more frequently reported a lack of time due to combined work and family duties, highlighting the dual demands faced by employed women [52].

## 6. Strategies to Improve Adherence

### 6.1. Age-Specific Interventions

A higher-level guide for low-cost fitness of older adults for the purpose of keeping good health, and preventing overuse, low-grade resistance exercise should also be available. Low-impact behaviors that should be suggested include the following: (1) Walking: Simple and easy to use, walking is the most powerful way to boost cardiovascular health, mental health and exercise levels and sleep and cognition. The CDC recommends at least 150 min of moderate-intensity activity per week. (2) Swimming: A full-body workout to reduce pressure on joints and build muscle strength and cardiovascular capacity. (3) Yoga and Tai Chi: This type of training boosts flexibility, balance, and meditation, which means it benefits your nerves; this practice also helps improve mental health. Chair yoga, for example, is especially advised for anyone who may have mobility issues. (4) Cycling—Both stationary and outdoor cycling provide aerobic benefits and are easy on the joints to develop muscle endurance and improve cardiovascular health. (5) Water Aerobics: Water aerobic exercise provides some resistance training, cardiovascular activity, and can offer joint support for a balance between resistance training and cardio [53].

Cognitive Training. Adding cognitive training to daily physical activity may be beneficial to older adults with memory damage. Such stimuli help boost cognitive function in people; some memory exercises or physical routines may bring mental vigilance and improve overall quality of life. Group-Based Programs. Group interventions that facilitate social interaction are vital in dealing effectively with the problem of isolation among older people. Programs emphasize social and physical activity together with programs contribute to the building of community support for such relationships and increase people’s habits and routine. Activities such as these create a sense of community, a necessary condition for mental health which involves not only social connection but also for people to participate in community-based programs in many places and therefore their mental well-being [53].

### 6.2. Gender-Specific Interventions

Flexible exercise plans are crucial to the lives of all women, but even more so for those who prioritize caregiving and household chores over other responsibilities. If programs offer women feasible ways to integrate physical activity into daily routines, this may influence both uptake and long-term adherence. For example, home-based sessions and brief, high-intensity formats may be more practical than longer endurance workouts for women with substantial caregiving and domestic responsibilities. It has been found that directed interventions designed to improve these limitations can substantially increase the global levels of physical activity among women, especially in aged 30 to 59 years [54].

Psychological barriers, such as depression and anxiety, can also lead to poor movement uptake in women. Prevention-based interventions (which include counseling or the provision of support networks) have been effective in maximizing motivation [55]. Also, making self-care practices a habit and taking care of one’s own well-being helps women overcome psychological barriers to performing physical activity. Furthermore, camaraderie and community building that female empowerment facilitates a more inclusive environment, and the environment promotes its social acceptability for adherence [54].

For men, a different approach can be more effective in motivating them to engage in physical activity. It is well known that performance-related anxieties run rampant in many men, and so making a point to stress the long-term health benefit of moderate exercise compared to a higher-intensity workout could go a long way to assuaging anxiety felt by some. Studies show that men, unlike women, focus mainly on enjoyable activities in their work routine. In light of this, emphasizing exercise as a rewarding, beneficial activity will make men feel more competent and less anxious, which will lead to higher participation [54].

### 6.3. Personalized Interventions

Tailoring the CR program to meet individual patient needs has been shown to significantly improve adherence. Conversely, understanding the barriers and motivational factors can assist in customizing CR programs to better suit individuals. This can include personalized communication strategies, such as sending motivational text messages to reinforce participation. Platforms like Well-Beat utilize psychological models to assist patients in coping with stress and staying engaged with the CR process. Personalizing exercise prescriptions to each patient’s abilities and health status can improve engagement and adherence [52].

Motivation to adhere and CR can be categorized into two main groups: beliefs and group cohesion, with a focus on support networks as well. Patients’ beliefs regarding the effectiveness and significance of CR are crucial for their participation. A strong belief in the health benefits of CR can enhance dedication to the program, as demonstrated in qualitative studies where participants emphasized the perceived importance of CR for their recovery [52].

### 6.4. Education and Counseling

Providing education about the benefits of CR, along with dietary counseling and exercise training, is crucial for patient compliance. For example, in lifestyle counseling, it is useful to also consider caffeine consumption: a recent review highlights that the relationship between coffee/caffeine and atrial fibrillation is complex and can vary by gender, suggesting personalized indications in counseling [56]. Encouraging patients to incorporate physical activity into their daily lives outside of formal CR sessions significantly improves long-term adherence [57]. Structured educational programs can lead to improved knowledge, which is associated with better adherence to lifestyle modifications. Evidence suggests that participation in educational sessions can enhance motivation and understanding of health behaviors. Group-based exercise fosters a supportive environment and encourages social interaction among participants. The camaraderie and shared experiences within a group can boost motivation and reduce feelings of isolation, making participants more likely to continue [52]. Many participants report that exercising in a group setting increases motivation and lowers the likelihood of dropping out due to mutual encouragement.

### 6.5. Addressing Barriers to Participation

Identifying and minimizing barriers to participation, such as transportation issues, scheduling conflicts, and financial constraints, is essential. Solutions may include flexible scheduling, telehealth options, and providing transport support for patients [58,59]. Improving the referral rates to CR programs by healthcare providers ensures that eligible patients receive timely information and resources to participate in CR. Educating providers about the importance of CR can enhance patient referrals and attendance. Mobile applications and devices, such as smartphones and fitness trackers, have been shown to increase engagement in CR by enabling real-time monitoring and personalized feedback [60,61]. They help with regular follow-ups and maintain patient motivation and adherence. Studies suggest that patients using mobile health (mHealth) tools during CR attend more sessions, demonstrate better adherence, and achieve improved outcomes, such as greater exercise capacity and better weight management [62].

### 6.6. Behavioral and Psychological Support

CR is a structured program recommended for individuals recovering from CVD. It combines exercise training, education on lifestyle changes, and psychosocial support to boost physical fitness, reduce cardiovascular risks, and improve overall quality of life [63]. There is a strong emphasis on both aerobic and resistance training, with benefits observed from combining the two [64]. Enhancing psychological support through counseling and behavioral interventions can help patients overcome anxiety and fear associated with physical activity. Programs that include mindfulness training and stress management can improve self-efficacy related to exercise. Addressing psychological factors such as anxiety and depression is crucial. Incorporating behavioral health counseling can help patients manage their emotional well-being and strengthen their adherence to CR programs [64].

The role of support systems is crucial. Social support from family, friends, and healthcare providers has a significant impact on adherence to CR programs. Research indicates that patients who receive encouragement from their family members or have positive interactions with rehabilitation staff are more likely to adhere to their CR plans [53]. Verbal encouragement and reassurance from rehabilitation staff can empower patients, increasing their confidence to engage in exercise routines [3].

**Figure 3:**
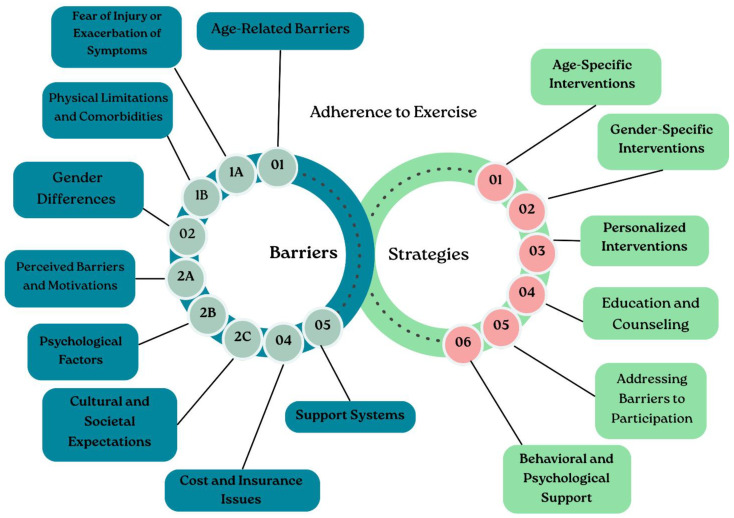
Graphical representation of the main barriers to exercise adherence in cardiac rehabilitation programs and the corresponding strategies to overcome them.

The summary of barriers and strategies is shown in Figure 3.

## 7. Conclusions

Sticking to exercise CR is crucial for secondary prevention in CVD. It has been shown to significantly reduce cardiac mortality and improve quality of life. However, in practice, referral, uptake, and completion rates are often suboptimal, especially among women, resulting in only partial realization of the benefits of CR.

The main barriers identified in studies are consistent and include physical limitations (such as fatigue, multimorbidity, and deconditioning), a lack of understanding of risk and CR benefits, financial constraints, and limited access.

Gender-specific barriers, such as caregiving responsibilities, lower referral rates, and societal expectations, make it particularly challenging for women to participate in CR programs. Promising strategies include personalized exercise plans, multidisciplinary care, and the use of technology (such as apps and remote monitoring) to improve access and support long-term engagement. Involving family and social networks can also enhance adherence, provided that issues like role conflict and competing responsibilities are addressed. In clinical practice, it is important to assess and address age- and gender-specific barriers to CR participation; increase referral and rates among women; and implement flexible, technology-driven, and family-inclusive care models. In research, there is a need to test interventions that are responsive to gender, evaluate long-term adherence and outcomes, and develop sustainable implementation strategies for different healthcare systems.

## Figures and Tables

**Table 1:** Sex-related patterns in referral, barriers, psychosocial factors and outcomes in exercise-based cardiac rehabilitation.

Domain	Men	Women
Referral and participation	More often referred to CR Higher enrolment and completion	Less often referred Lower enrolment and completion rates
Main barriers	Work and time constraints logistics (travel, schedule) Lower motivation/interest	Caregiving and family duties Lack of time for self-care Lower exercise self-efficacy
Phsyicological and social factors	Pressure to perform Work-related stress	Higher depression and anxiety Social norms prioritizing family over their own health
Response to CR and outcomes	Greater gains in VO_2_ and functional capacity Better prognosis when adherent	Slightly smaller VO_2_ gains but similar prognostic benefit when adherent Poor adherence linked to worse outcomes and recurrent events

## Data Availability

No new data were created or analyzed in this study. Data sharing is not applicable to this article.
